# Early Life Stress-Induced Epigenetic Programming of Hippocampal NPY-Y2 Receptor Gene Expression Changes in Response to Adult Stress

**DOI:** 10.3389/fncel.2022.936979

**Published:** 2022-07-01

**Authors:** Derya Kocamaz, Caroline Franzke, Nicole Gröger, Katharina Braun, Jörg Bock

**Affiliations:** ^1^Department of Zoology/Developmental Neurobiology, Institute of Biology, Otto von Guericke University Magdeburg, Magdeburg, Germany; ^2^Center for Behavioral Brain Sciences, Magdeburg, Germany; ^3^PG “Epigenetics and Structural Plasticity,” Institute of Biology, Otto von Guericke University Magdeburg, Magdeburg, Germany

**Keywords:** DNA-methylation, hippocampus, Y2 receptor (Y2R), maternal separation, epigenetic, resilience

## Abstract

Early Life Stress (ELS) can critically influence brain development and future stress responses and thus represents an important risk factor for mental health and disease. Neuropeptide Y (NPY) is discussed to be a key mediator of resilient vs. vulnerable adaptations and specifically, the NPY-Y2 receptor (Y2R) may be involved in the pathophysiology of depression due to its negative regulation of NPY-release. The present study addressed the hypotheses that ELS and adult stress (AS) affect the expression of hippocampal Y2R and that exposure to ELS induces an epigenetically mediated programming effect towards a consecutive stress exposure in adulthood. The specific aims were to investigate if (i) ELS or AS as single stressors induce changes in Y2 receptor gene expression in the hippocampus, (ii) the predicted Y2R changes are epigenetically mediated *via* promoter-specific DNA-methylation, (iii) the ELS-induced epigenetic changes exert a programming effect on Y2R gene expression changes in response to AS, and finally (iv) if the predicted alterations are sex-specific. Animals were assigned to the following experimental groups: (1) non-stressed controls (CON), (2) only ELS exposure (ELS), (3) only adult stress exposure (CON+AS), and (4) exposure to ELS followed by AS (ELS+AS). Using repeated maternal separation in mice as an ELS and swim stress as an AS we found that both stressors affected Y2R gene expression in the hippocampus of male mice but not in females. Specifically, upregulated expression was found in the CON+AS group. In addition, exposure to both stressors ELS+AS significantly reduced Y2R gene expression when compared to CON+AS. The changes in Y2R expression were paralleled by altered DNA-methylation patterns at the Y2R promoter, specifically, a decrease in mean DNA-methylation in the CON+AS males compared to the non-AS exposed groups and an increase in the ELS+AS males compared to the CON+AS males. Also, a strong negative correlation of mean DNA-methylation with Y2R expression was found. Detailed CpG-site-specific analysis of DNA-methylation revealed that ELS induced increased DNA-methylation only at specific CpG-sites within the Y2R promoter. It is tempting to speculate that these ELS-induced CpG-site-specific changes represent a “buffering” programming effect against elevations of Y2R expression induced by AS.

## Introduction

Early life stress (ELS) is a critical risk factor underlying the etiology of mental disorders since it critically affects the development of neuronal circuits in limbic and prefrontal cortical brain areas (Bock et al., 2014; Babenko et al., 2015; Burkholder et al., 2016; Birnie et al., 2020). Repeated maternal separation in rodents is a widely used experimental paradigm to investigate the behavioral, physiological, and cellular/molecular consequences of ELS (Groger et al., 2016). So far, the majority of studies have focused on the detrimental outcome of ELS and described the emergence of psychopathological behaviors including depressive-like behavioral symptoms (Dimatelis et al., 2016; Sadeghi et al., 2016; Tractenberg et al., 2016; Tan et al., 2017; Pena et al., 2019; Bachiller et al., 2020). However, there is increasing evidence that ELS can also induce adaptive neuronal and cellular changes that promote stress resilience and enhance the flexibility of behavioral adaptations to challenging encounters later in life (Zannas and West, 2014; McEwen, 2016; Santarelli et al., 2017; Agorastos et al., 2019; Hodes and Epperson, 2019). Furthermore, the analysis of sex-specific differences has not yet been addressed in much detail, although more recent literature reveals that the long-lasting consequences of ELS on the brain and behavior are sex-dependent (Bonapersona et al., 2019).

A more detailed mechanistic understanding of the long-term consequences of ELS and how ELS exposure may affect stress responses and behavior later in life can only be obtained by exposing individuals to multiple and also different types of stressors at different life spans (Orso et al., 2020). This led to the “two-(or multiple) hit” concept, which claims that early life adversities may exert a “programming” effect on the behavioral and on the cellular/molecular level and thereby increase or decrease stress sensitivity later in life (Lupien et al., 2009; Gruber et al., 2015; Lesse et al., 2017; Santarelli et al., 2017; Jaric et al., 2019). There is increasing evidence that specific epigenetic changes are underlying such programming effects induced by early life experiences (Bock et al., 2014; Groger et al., 2016). Epigenetic mechanisms are defined as alterations in gene expression levels that are not due to changes in the DNA sequence (Lacal and Ventura, 2018) and are considered the interface between early environmental influences and genetically programmed developmental processes in the brain. Thus, these mechanisms are also thought to play a crucial role in translating early adverse experiences into long-lasting gene expression changes that may underlie changes in stress-related behaviors and psychopathologies (Bock et al., 2014; Groger et al., 2016; Alexander et al., 2018; Alyamani and Murgatroyd, 2018; Li et al., 2020).

Neuropeptide Y (NPY), one of the most abundant neuropeptides in several brain regions such as the amygdala, hippocampus, and hypothalamus, is considered to be a key mediator in the development of vulnerability and resilience (Nahvi and Sabban, 2020). Besides other physiological functions such as regulating food intake, energy homeostasis, and circadian rhythms, there is evidence that NPY plays a complex role in the regulation of stress-affective behavior and stress resilience by suppressing the effects of stress-related neurotransmitters (Enman et al., 2015; Kautz et al., 2017; Hassan et al., 2019). In healthy subjects’ plasma NPY levels have been shown to rise in response to stress, whereas in depressed individuals’ plasma and CSF NPY levels are decreased when compared to healthy controls (Widerlov et al., 1988; Hashimoto et al., 1996; Nilsson et al., 1996; Heilig et al., 2004; Hou et al., 2006). Animal studies have shown that NPY knockout mice are more anxious and hippocampal or amygdala NPY overexpression renders animals less anxious (Bannon et al., 2000; Thorsell et al., 2000; Primeaux et al., 2005). The neuromodulatory effects of NPY are mediated by at least six G-protein coupled receptors, of which Y1, Y2, and Y5 are of the most important in behavioral studies. The functions and distribution of these receptors in the nervous system differ depending on brain regions (Duarte-Neves et al., 2016). In the mouse brain, the Y2 receptor (Y2R) shows a widespread distribution in cell bodies and nerve terminals, specifically in different hippocampal subfields, the amygdala, bed nucleus of stria terminalis, nucleus accumbens, and hypothalamus (Stanic et al., 2006). The Y2R is a presynaptic autoreceptor that negatively regulates the release of NPY, and thus its stimulation can exert anxiogenic effects (Chen et al., 1997; Redrobe et al., 2002; Reichmann and Holzer, 2016; Nwokafor et al., 2020). There is increasing evidence that alterations in the expression of this receptor subtype may be associated with stress-related disorders such as major depression and PTSD (Treutlein et al., 2017; Stone et al., 2020).

The aim of this study was to test the hypothesis that ELS induces long-term changes in DNA-methylation specifically at Y2R promoter sites, resulting in changes in Y2R expression that represent a programming effect for stress susceptibility in adulthood. Using a repeated maternal separation protocol in mice as an ELS paradigm (Lesse et al., 2017; Kohler et al., 2019), we analyzed if (i) ELS affects Y2R expression in the hippocampus at adulthood, (ii) if the proposed Y2R changes are epigenetically regulated *via* promoter-specific DNA-methylation, and (iii) if these ELS-induced “programming effects” of Y2R expression interfere with changes in Y2R expression and DNA-methylation in response to an adult stressor (forced swimming). In addition, we investigated if (iv) the stress-induced changes in Y2 receptor expression differ in the male and female hippocampus.

## Materials and Methods

### Animals

C57BL/6 mice (Janvier, Marseille, France) were housed on a 12:12 light/dark cycle with food and water provided *ad libitum*. Cages were not cleaned during the first two weeks after birth (defined as postnatal day 0; PND 0) to reduce handling stress. The nesting materials of home cages were changed once a week. The experimental protocols were approved by the ethics committee of the government of the state of Saxony-Anhalt according to the German guidelines for the care and use of animals in laboratory research (§8 TSchG; AZ: 42502–2-1272).

### Experimental Design

#### Stress Paradigms

##### Early Life Stress (ELS)

Early life stress was induced by maternal separation from PND 1 to PND 21: pups were removed from the home cage and individually isolated in boxes (13 × 13 cm) for 3 h (09:00–12:00 am), which allowed olfactory and auditory but no visual or body contact with their separated siblings. During the first week, the isolation boxes were placed in a humidified incubator (32°C) to create optimum conditions for the pups. Dams were kept in their home cages during the separation period. Prior to the return of the pups, half of the nesting material was removed and replaced by new nesting materials to increase maternal stress and stimulate nest building after the pups were returned. In the afternoon following the last separation on PND21, the pups were divided by litter size and subsequently kept together in groups with 3–5 animals of the same sex until the time of the respective experiment.

##### Adult Stress (AS)

Adult stress was induced by exposing animals to swim stress on two consecutive days (PND 114–115). Animals were forced to swim for 15 min each day in a glass tank containing water (22°C).

#### Experimental Groups

**FIGURE 1:**
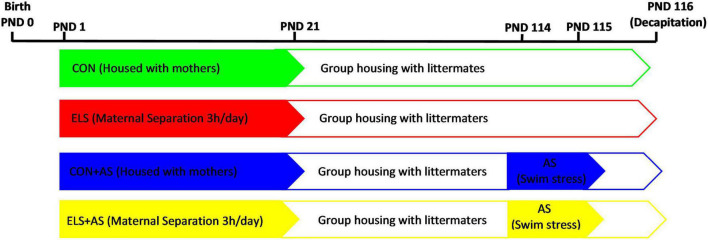
Experimental design.

The animals were assigned to the following groups (Figure 1).

Control (CON): Animals of this group were not exposed to any stressors (*N*_*male*_:8; *N*_*female*_:10) and were housed together with their mother until PND 21. After PND 21, they were weaned and subsequently housed in groups with up to 3–5 animals of same sex until the time of the respective experiment.

Early life stress: Animals of this group were exposed to maternal separation, after weaning they were reared as the control animals. (*N*_*male*_:10; *N*_*female*_:10).

CON+AS: Animals of this group were reared as the controls but were exposed to adult stress at PND 114 and 115 (*N*_*male*_:9; *N*_*female*_:9).

ELS+AS: Animals of this group were reared as described above but exposed to both stressors, ELS and AS (*N*_*male*_:9 *N*_*female*_:9).

### Gene Expression Analysis

Animals were sacrificed by decapitation on PND 116. Brain tissue of the right and left hippocampus was collected, immediately frozen in liquid nitrogen, and stored at −80°C until analysis. Total RNA from the right hippocampus was extracted using the innuPREP RNA Mini Kit 2.0 (Analytik Jena, Berlin, Germany) according to the manufacturer’s instructions. The genomic DNA contaminants were removed using the innuPREP DNase I Digest Kit (Analytik Jena, Berlin, Germany). One-step quantitative real-time PCR was performed using the Rotor-Gene Multiplex RT-PCR Kit (QIAGEN GmbH, Hilden, Germany) and TaqMan gene expression assays (Life Technologies, Carlsbad, California, United States). Commercially available mouse Taqman probes were used for Y2 receptor (Mm01218209_m1; FAM) as a target gene and hypoxantine guanine phosphoribosyl transferase (Hprt, Mm00446968_m1; VIC) as a reference gene. For gene assays, all samples were run in triplicate. The analysis was replicated by an independent second real-time PCR. The relative gene expression of each sample was calculated using the delta-delta CT method after normalization using the reference gene.

### DNA-Methylation Analysis

**FIGURE 2:**
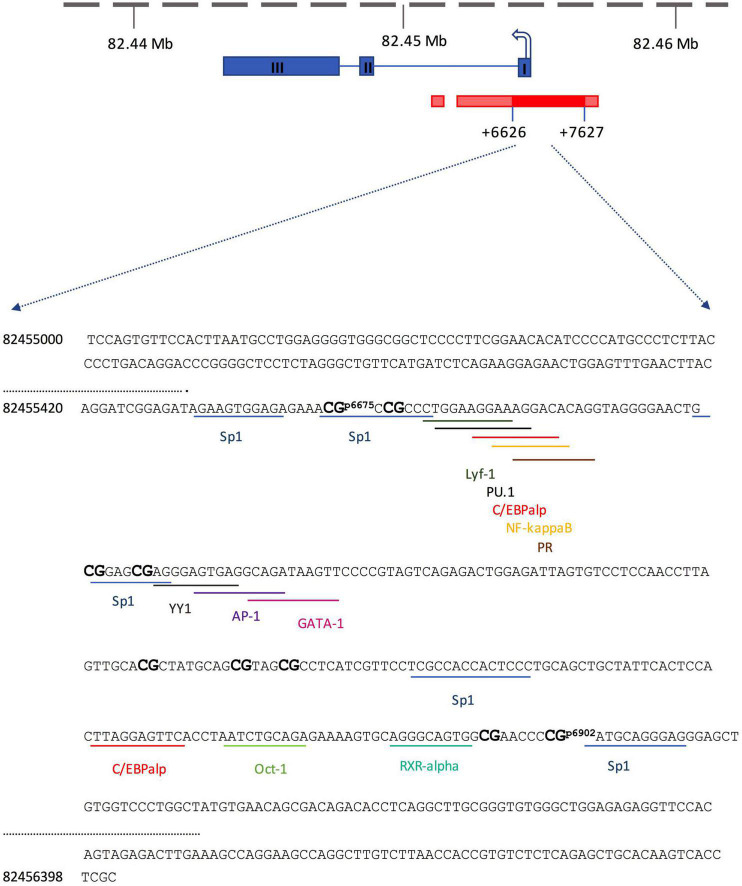
Schematic depiction of the mouse Y2 receptor gene, including the positions of the 9 analyzed CpG sites and the transcription factor binding sites (blue: exon; dark red: core promoter; red: promoter bounds and flanking region). The sequences of the promoter region are shown as a forward strand (NCBI, Gene ID: 18167).

Genomic DNA was extracted from the left hippocampus using the DNeasy Blood & Tissue Kit (QIAGEN GmbH, Hilden, Germany) with QIACube (QIAGEN GmbH, Hilden, Germany) according to the manufacturer’s instructions. The bisulfite modification of genomic DNA was carried out by using the EpiTect Bisulfite kit (QIAGEN, Hilden, Germany), and the cleanup of bisulfite-converted DNA was automatically done with QIAcube (QIAGEN, Hilden, Germany) following to the manufacturer’s protocol. The DNA-methylation levels were analyzed using the methods of bisulfite PCR and pyrosequencing. The DNA sequence of the Y2 receptor gene was identified using National Center for Biotechnology Information (NCBI) and the promoter region was defined using the Ensembl Genome Browser (Figure 2). The genomic locations of each CpG-site were determined according to their distances from the start codon. Four primers covering an overall of nine CpG-sites in the promoter region of the Y2 receptor gene were designed using PyroMark Assay Design software (QIAGEN, Hilden, Germany) and confirmed to give specific PCR products by capillary electrophoresis (Table 1). The biotinylated PCR product was purified using streptavidin sepharose beads (GE Healthcare, Milwaukee, WI, USA) and Pyrosequencing Vacuum Prep Tool (QIAGEN, Hilden, Germany). Pyrosequencing was performed with Pyromark Q96 ID (QIAGEN, Hilden, Germany) using Pyromark Q96 Gold reagents (QIAGEN, Hilden, Germany) according to the manufacturer’s instructions. The CpGenome Universal Methylated mouse genomic DNA (Millipore, Darmstadt, Germany) was used as a positive control to verify bisulfite conversion and methylation analyzes, while the negative control was self-made in our laboratory using the REPLI-g mini kit (QIAGEN, Hilden, Germany) according to the manufacturer’s protocol. All samples for DNA-methylation were run in duplicate, and samples that did not pass the quality control test were repeated. Pyrosequencing results were automatically analyzed using PyroQ-CpG software (Qiagen). For predicted transcription factor binding sites within the mouse Y2 receptor gene promoter region, the Alibaba2.1 software was used (Grabe, 2002).

**TABLE 1:** Designed Y2 receptor gene primers used for DNA-methylation.

	Chromosomal location	Distance from the start codon	
*P1*	Chr3:82455447-82455477	p6675–p6678	Forward primer GGAGGAGGATAGGAGATAGAAGT Reverse primer (biotinylated) CAATTCCCCTACCTATATCCTTT Sequencing primer AGATAGAAGTGGAGAGA
*P2*	Chr3:82455487-82455512	p6713–p6718	Forward primer GTTTGGAAGGAAAGGATATAGGT Reverse primer (biotinylated) AAACACTAATCTCCAATCTCTAACT Sequencing primer ATAGGTAGGGGAATTG
*P3*	Chr3:82455562-82455584	p6788–p6798-p6803	Forward primer AGGGAGTGAGGTAGATAAGTTT Reverse primer (biotinylated) ACTACCCTACAACTTTTCTCTACA Sequencing primer AGTGTTTTTTAATTTTAGTTGTA
*P4*	Chr3:82455669-82455701	p6895–P6902	Forward primer GATTGGAGATTAGTGTTTTTTAATTTTAGT Reverse primer (biotinylated) TATTCACATAACCAAAAACCACAACTC Sequencing primer AGTGTAGGGTAGTGG

### Statistics

A two-way analysis of variance (ANOVA) with the main factors ELS and AS and a *post hoc* Student Newman–Keuls (SNK) test were performed to determine differences between the four experimental groups regarding gene expression, mean DNA-methylation, and CpG-site-specific DNA-methylation (*p* ≤ 0.05 considered significant). In addition, Pearson correlation analysis was used to calculate the correlation between Y2 receptor gene expression and mean DNA-methylation. All statistical analyzes were performed using GraphPad Prism 7.0 software and all data were presented as mean value ± SD. The significance value is indicated in the figures as ^∗^*p* ≤ 0.05. For estimating the sample power (1-β err prob) of the analyzes, we first calculated the effect size (Cohen’s *f*) for the respective ANOVAs and the pooled SD (σpool) of the different groups. With these parameters, a power analysis was performed using G^∗^Power 3.1.9.7^1^.

## Results

### Y2 Receptor Gene Expression in the Hippocampal Formation

#### Males

**FIGURE 3:**
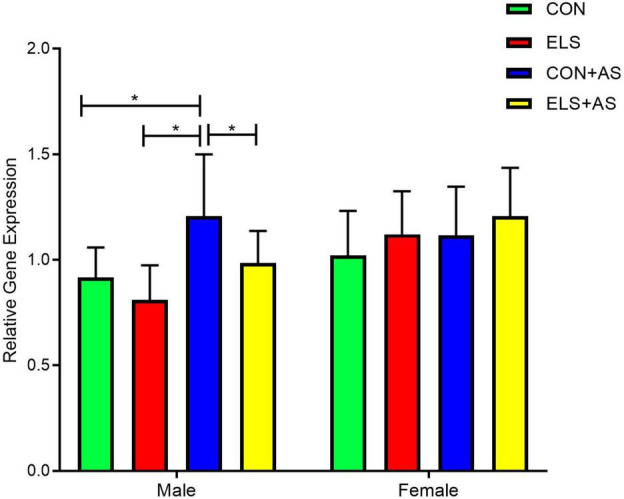
Analysis of Y2 receptor gene expression after ELS and AS in the hippocampus of male and female animals. Significant results of *post hoc* test (SNK) are represented as *p ≤ 0.05. CON, control; ELS, early life stress; CON+AS, control + adult stress; ELS+AS, early life stress + adult stress.

In male animals, two-way ANOVA (1-β err prob = 0.98) revealed a significant main effect for ELS (*p* = 0.0179) and for AS (*p* = 0.0013) but no interaction between the two factors (*p* = 0.3916, Figure 3). Overall, animals from the AS groups displayed significantly higher gene expression than NAS animals. Also, the animals from the control groups had higher gene expression than the animals from the ELS groups.

The *post hoc* test for individual group comparisons revealed significantly increased gene expression levels in the CON+AS animals when compared to the CON and ELS group as well as to the ELS+AS animals (Figure 3, *p* ≤ 0.05). No significant difference was detected between the unstressed controls, the ELS, and the ELS+AS groups.

#### Females

In female animals, two-way ANOVA (1-β err prob = 0.49) revealed no significant main effects for ELS (*p* = 0.785) nor for AS (*p* = 0.0915) and no interaction between the two factors (*p* = 0.2781).

### DNA-Methylation Analysis

To test for a proposed epigenetic regulation of the detected gene expression changes in male animals, DNA-methylation changes in nine CpG-sites within the promoter region of the Y2 receptor gene were examined. First, we analyzed and compared the mean DNA-methylation over the nine CpG-sites (mean DNA-methylation). In addition, a more detailed analysis and comparison of DNA-methylation levels at each individual CpG-site (CpG-site-specific analysis) were conducted.

#### Mean DNA-Methylation of the 9 Analyzed CpG-Sites Within the Y2 Receptor Promoter in Male Animals

**FIGURE 4:**
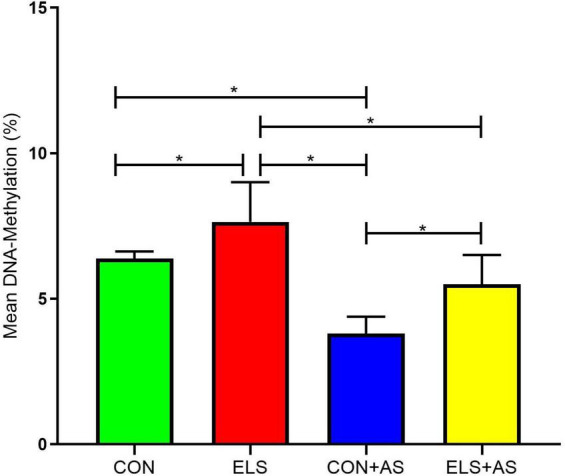
Mean DNA-methylation in the Y2 receptor gene promoter of male animals. Significant results of *post hoc* test (SNK) are represented as *p ≤ 0.05. CON, control; ELS, early life stress; CON+AS, control + adult stress; ELS+AS, early life stress + adult stress.

Two-way ANOVA (1-β err prob = 1.0) showed a significant main effect for ELS (*p* ≤ 0.0001) and AS (*p* ≤ 0.0001) but no interaction between the two factors (*p* = 0.538, Figure 4). Overall, animals from the AS groups displayed significantly lower mean DNA-methylation than the animals, that did not experience AS. Also, the animals from the control groups showed reduced mean DNA-methylation when compared to animals exposed to ELS.

A *post hoc* test revealed a significantly increased mean DNA-methylation in animals from the ELS group when compared to the CON group, the CON+AS group, and the ELS+AS group (Figure 4, *p* ≤ 0.05). In addition, CON+AS animals showed significantly decreased mean DNA-methylation when compared to the ELS+AS group and to unstressed controls (*p* ≤ 0.05). No significant changes were detected between the ELS+AS and the CON groups.

#### Correlation With Y2 Receptor Gene Expression

**FIGURE 5:**
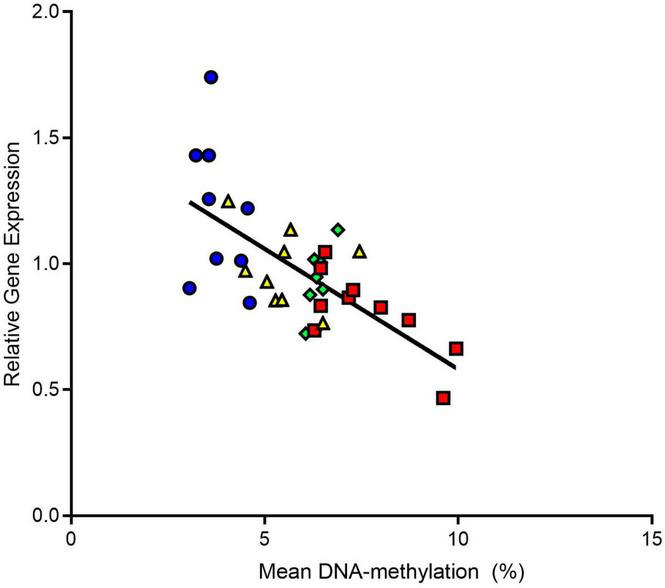
Correlation analysis between Y2 receptor gene expression and mean DNA-methylation overall male experimental groups. We found that Y2 gene expression was negatively correlated to mean DNA-methylation. CON, control; ELS, early life stress; CON+AS, control + adult stress; ELS+AS, early life stress + adult stress.

A Pearson correlation analysis overall experimental groups in males revealed a strong negative correlation between mean DNA-methylation and Y2R gene expression (*r*: -0,6728; *p* ≤ 0.0001, Figure 5).

#### CpG-Site-Specific DNA-Methylation Analysis Within the Y2 Receptor Gene Promoter in Male Animals

**FIGURE 6:**
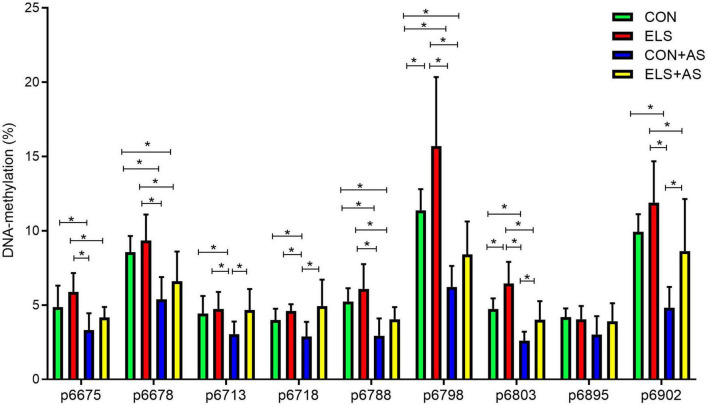
Changes of DNA-methylation (%) at analyzed CpG sites within the Y2 receptor gene promoter in male animals. The locations of CpG-sites were determined according to their distance from the start codon. Significant results of the *post hoc* test (SNK) are represented as *p ≤ 0.05. CON, control; ELS, early life stress; CON+AS, control + adult stress; ELS+AS, early life stress + adult stress.

In principle, two-way ANOVA for each CpG-site indicated similar effects as described for mean DNA-methylation, but not all CpG-sites were affected in the same manner (see Table 2, also for sample Power). The CpG-site-specific effects are also clearly indicated by the results of the *post hoc* test, revealing that ELS when compared to unstressed controls resulted in significantly enhanced DNA-methylation only at two circumscribed CpG-sites, p6798 and p6803 (*p* ≤ 0.05, Figure 6). In contrast, when comparing CON+AS animals with the CON and ELS groups decreased DNA-methylation was found at all investigated CpG-sites, except p6895 (*p* ≤ 0.05). Compared to the ELS+AS, the CON+AS animals decreased DNA-methylation was only found at CpG-sites p6713, p6718, p6803, and p6902 (*p* ≤ 0.05). In addition, in ELS+AS animals decreased DNA-methylation was determined at CpG-sites p6678, p6788, and p6798 when compared to unstressed control animals and at p6675, p6678, p6788, p6798, p6803, and p6902 CpG-sites when compared to the ELS group (*p* ≤ 0.05).

**TABLE 2:** Two-way ANOVA results for individual CpG site-specific DNA-methylation within the Y2 receptor gene promoter in male animals.

Individual CpG site	Main effect		Interaction	Sample power
	ELS	AS	ELS*AS	1-β err prob
*p6675*	Yes	Yes	No	0,99
*p6678*	No	Yes	No	0,99
*p6713*	Yes	No	No	0,85
*p6718*	Yes	No	Yes	0,96
*p6788*	Yes	Yes	No	0,99
*p6798*	Yes	Yes	No	0,99
*p6803*	Yes	Yes	No	0,99
*p6895*	No	No	No	0,64
*p6902*	Yes	Yes	No	0,99

## Discussion

The “Developmental Origins of Health and Disease” (DOHaD) concept postulates that distinct environmental influences, such as early life stress, occurring during critical phases of development “program” the development of an individual and thereby influence health and susceptibility to disease later in life (Gluckman and Hanson, 2006). In support of this concept, our results show that ELS exerts a programming effect towards the upregulation of Y2 receptor expression in response to adult stress in a sex-specific manner. Additionally, our results reveal that this ELS-induced programming effect appears to be at least in part mediated *via* changes in DNA-methylation.

### Effects of Early Life Stress and Adult Stress on Hippocampal Y2 Receptor Gene Expression

Pre- and postnatal exposures to ELS are major programming factors for neuronal and behavioral development and thus represent a risk factor underlying the etiology of mental disorders (Heim and Nemeroff, 2001; Bock et al., 2014, 2015; Babenko et al., 2015; Braun et al., 2020). Although the majority of experimental studies focused on stress vulnerability and pathological behavioral outcomes, evidence is mounting that acute, brief, or intermittent ELS can also promote neuronal and cellular adaptations to changing or threatening environments, resulting in improved stress coping and resilience in later life (Xie et al., 2013; Zannas and West, 2014; McEwen, 2016; Santarelli et al., 2017). However, the neuronal and cellular mechanisms mediating and determining stress vulnerability or resilience are still poorly understood. There is increasing evidence that the neuropeptide Y, one of the most abundant neuropeptides in the brain with particularly high expression in the hippocampus and cortical areas, may function as an endogenous mediator of pathological or resilient adaptations after ELS (Dumont and Quirion, 2006; Enman et al., 2015; Kautz et al., 2017; Hassan et al., 2019). A negative regulator of NPY release is the presynaptic Y2 receptor. Alterations in the expression of this receptor subtype are associated with stress-related disorders such as major depression and PTSD (Treutlein et al., 2017; Stone et al., 2020).

In the present study, we first focused on changes in the expression of the Y2 receptor gene in response to ELS and in response to AS. At the gene expression level, we found that ELS alone did not significantly change Y2 receptor gene expression in adult males and females. However, in rats, it was reported that the NPY-system is sensitive towards ELS since it was reported that NPY-like immunoreactivity in the hippocampus and basolateral amygdala was decreased after exposure to maternal separation stress (Jimenez-Vasquez et al., 2001; Miragaia et al., 2018). In contrast, we found that exposure only to AS increased Y2 receptor gene expression in male animals. Presynaptic Y2 receptors are negative regulators of NPY release, e.g., it was shown that deletion or blockade of Y2 receptor elevates NPY levels, which can exert antidepressant-like effects (Tschenett et al., 2003; Carvajal et al., 2006). Accordingly, the AS-induced upregulation of Y2 receptors observed in males may result in reduced NPY levels and thereby confer an anxiogenic effect. The stress-induced changes in Y2 receptor gene expression in the hippocampus found in our study were only evident in male animals but not in females. So far, only a few studies have systematically focused on sex differences with respect to individuals exposed to ELS, which is surprising since a considerable sex-bias in the prevalence of ELS-induced disorders has to be considered. A number of studies indicate that the sex of the offspring represents an important preprogrammed genetic factor for the consequences of early life adversities and the development of vulnerability or resilience in later life periods (Bock et al., 2011; Kunzler et al., 2015; Goodwill et al., 2019; Bath, 2020). However, literature on this topic is still quite controversial and does not yet reveal a clear picture of sex-specific effects since sex-bias is not consistent in terms of the direction of the outcome (reviews (Dalla et al., 2011; Bock et al., 2014; Seney and Sibille, 2014). Nevertheless, some of the findings of recent studies are in line with the findings of our study. For example, using high-resolution diffusion magnetic resonance imaging, it was shown that exposure to ELS in mice induced fronto-limbic hyper-connectivity in males, but either no change or hypoconnectivity in females (White et al., 2020). Also, it was shown in C57BL/6 mice that ELS by maternal separation and restraint stress around puberty increased depression-like behavior only in males but not in females. Only a combination of both stressors increased depression-like behavior in both sexes (He et al., 2020). In line with these findings, it was shown that maternal separation in mice affected stress-physiology and anxiety-like outcomes only in males, whereas females remained unaffected. A similar finding was reported in rats where it was shown that females were resistant to developing a depressive phenotype after maternal separation (Dimatelis et al., 2016). Although far from understood, increasing evidence indicates that the differences between males and females in response to ELS or adult stress are the result of a sexual dimorphic development that leads to sex-differences in brain, behavior, and stress systems such as the HPA-axis (Bath, 2020). Sexual hormones, androgens and estrogens, are thought to play a major role in these developmental processes and may also be involved in the stress response. Although it is unlikely that sexual hormones are directly affected during ELS (maternal separation), one has to consider that the brain undergoes a substantial reorganization and differentiation during adolescence, which is critically influenced by gonadal hormones. Since in our study, adult animals are investigated, we cannot exclude that changes in gonadal hormones during adolescence may affect the differential outcome between males and females in our study. Estrogens may also play a role in the stress response in adulthood. For females, it has been shown that the estrous cycle regulates dendritic spine turnover in the hippocampus and that stress-induced reduction in dendritic spine density is dependent on the presence of estrogen (Shors et al., 2001; McEwen et al., 2015). Gonadal hormones can also influence HPA-axis regulation and thereby affect the development of the stress response in later life (Panagiotakopoulos and Neigh, 2014). The development of the HPA-axis is also critically influenced by maternal care. Therefore, it is of interest that sex-specific differences in maternal care directed to male or female offspring are reported (Bath, 2020). Thus, it is tempting to speculate that sex-specific differences in maternal care during our ELS period may also contribute to the detected differences between males and females in this study.

### Programming Effects of Early Life Stress on Adult Stress -Induced Changes in Y2 Receptor Gene Expression

Another aim of this study was to address the hypothesis that ELS might exert a programming effect on the observed upregulation of Y2 receptor gene expression in response to AS exposure. In ELS pre-exposed male mice with additional exposure to AS (ELS+AS group), hippocampal Y2 receptor gene expression remained at the level of unstressed controls and was significantly lower when compared to the AS only group. Hence, ELS pre-exposure appears to “buffer” against Y2R upregulation in response to AS and thereby may protect the animal from the negative consequences of AS by promoting stress coping (resilience). In view of the observed changes in DNA-methylation (see below), it is tempting to speculate that ELS programs AS-induced Y2 receptor gene upregulation *via* epigenetic mechanisms. A similar “programming” effect of ELS towards effects of AS exposure on gene expression has also been observed in a previous study on hippocampal oxytocin receptor (OxtR) and arginine vasopressin receptor type 1a (AvpR1a) gene expression (Lesse et al., 2017). In this study, we found that ELS or AS alone had no effect on gene expression, whereas ELS pre-exposed animals displayed elevated hippocampal OxtR gene expression in response to AS exposure. The findings of our studies are also in line with other investigations on the effects of multiple/consecutive stressors (two- or multiple-hit concept), which found different gene expression patterns in the hippocampus of stress-naïve animals in response to a single stressor when compared to animals, that were exposed to multiple stressors (Gray et al., 2014). Various studies, including our own, also show that vulnerable and resilient adaptations may occur depending on genetic predispositions and the type, duration, and time point of stress exposure (Nederhof and Schmidt, 2012; Daskalakis et al., 2013; Mc Elroy and Hevey, 2014). With regard to the findings of the present study on Y2R expression, the question arises what are the factors that are contributing to a potential “protective” effect of ELS exposure. An important concept within this context is “social buffering” stating that the presence and interaction with conspecifics can attenuate the stress response and as a long-term consequence, may help to promote adaptive processes influencing responses to adverse experiences in later life periods (Beery and Kaufer, 2015; Gunnar, 2017; Yirmiya et al., 2020; Avellaneda and Kamenetzky, 2021). In early life, an important social buffering factor is the maternal care behavior of the mother, while in later life, interaction with same age conspecifics, for example, littermates, provides an important environmental influence. Previous studies have revealed that stimulation of early postnatal maternal care behavior by handling can reverse or restore disturbed neurodevelopmental mechanisms and dysfunctional behavior induced by prenatal stress (Bock et al., 2011; Weinstock, 2017). For the present study, we cannot exclude that the lack of maternal care during the 3 h separation periods may be compensated by higher levels of maternal behavior upon reunion after separation or during the normal housing conditions for the rest of the day, thus contributing to the described effects. However, it appears unlikely that the applied ELS paradigm in this study stimulates specific maternal care behavior that promotes the proposed protective effects, since applying the identical ELS paradigm resulted in enhanced stress sensitivity and increased depressive-like behavior in previous studies (Lesse et al., 2017; Kohler et al., 2019). In contrast to these studies, where animals were reared in social isolation after weaning, in the present study, animals were housed socially in same-sex groups with up to 3–5 animals until the time of the respective experiment. Thus, it is tempting to speculate that these postweaning and post-stress social rearing conditions provide a social buffering factor, stimulating protective adaptations. However, future experiments are required to analyze the critical role of post-stress rearing conditions for adaptive or maladaptive processes after ELS in more detail.

### Stress-Induced Changes in Hippocampal Y2 Receptor Gene Expression Are Mediated *via* Promoter-Specific DNA-Methylation

There is increasing evidence that the programming effect of ELS on the brain and behavioral functions is mediated *via* epigenetic mechanisms (Alyamani and Murgatroyd, 2018; Li et al., 2020). Among epigenetic changes with respect to early-life programming, DNA-methylation represents one of the best-understood mechanisms. This epigenetic modification represents a crucial regulator for the fine-tuning of gene expression, and the classical view is that enhanced DNA-methylation (hypermethylation) leads to decreased or even silenced gene expression. DNA-methylation can regulate and mediate the effects of a temporary exposure to early adverse situations into stable alterations in gene expression after the initial exposure has ended (Nuyt and Szyf, 2007).

Thus, another aim of this study was to address the hypothesis that the stress-induced alterations of Y2 receptor expression in response to ELS, AS, and to both consecutive stressors are epigenetically regulated *via* changes in DNA-methylation, specifically at the promoter region of the Y2 receptor gene. In support of this hypothesis, we found that the stress-induced changes in hippocampal Y2 receptor gene expression in male animals were accompanied by changes in DNA-methylation at the Y2 receptor gene promoter. Mean DNA-methylation over the 9 analyzed CpG-sites was affected by ELS (higher DNA-methylation) and by AS (lower DNA-methylation) indicating that the detected changes in Y2 receptor gene expression are at least partly mediated *via* altered DNA-methylation. This association is further supported by the strong negative correlation of gene expression with mean DNA-methylation observed overall experimental groups. Moreover, in line with the findings on the gene expression level, we found additional support for the ELS programming hypothesis. Our CpG-site-specific analysis revealed that ELS exposure alone induced increased DNA-methylation only in two circumscribed CpG sites when compared to unstressed controls. In contrast, exposure only to adult stress (C+AS) resulted in decreased DNA-methylation at nearly all analyzed CpG-sites, when compared to unstressed controls. This effect was partly blocked by prior exposure to ELS. Taken together and as already outlined above, it is tempting to speculate that these subtle, well-defined CpG-site-specific changes in DNA-methylation may reflect a possible mechanism underlying ELS-induced long-term “programming” and thereby protect the ELS pre-exposed animals against “anxiogenic” Y2 receptor changes in response to AS.

With respect to the interpretation of our data, one has to keep in mind that the regulation of gene expression is not only regulated by DNA methylation alone but also is orchestrated by a complex interaction with other epigenetic mechanisms such as posttranslational modifications of histones (Rothbart and Strahl, 2014). Several studies have reported that DNA methylation and histone modifications are mechanistically interdependent and play key roles in mediating the effects of environmental influences (Vaissiere et al., 2008; Ciccarone et al., 2018; Lee et al., 2020). Epigenetically mediated changes in gene expression are also associated with inhibited or accelerated actions of specific transcription factors at the respective CpG sites in gene promoter regions. The region of our analyzed CpG-sites in the Y2 receptor promoter covers short-specific DNA sequence motifs for the transcription factor (SP1). SP1 is involved in the control of several processes such as cell growth, apoptosis, synaptic plasticity, stress, and immune response, and importantly, it is also involved in the regulation of NPY (Wernersson et al., 1998).

## Conclusion

Our results reveal that early adverse experiences such as ELS and adult stress exposure affect the expression of Y2 receptors in the hippocampus of male mice, which at least in part are epigenetically mediated *via* changes in DNA-methylation. Moreover, our findings indicate that ELS can act as a “protective” programming factor towards the responses to subsequent stress experiences in adulthood. This programming effect appears to be mediated by subtle alterations in DNA-methylation at specific CpG-sites. However, future studies are required to assess the described consequences of single vs. multiple stress exposures in more detail, particularly if the observed changes of Y2 receptor expression may result in pathological or resilient behavioral phenotypes in later life.

## Data Availability Statement

The original contributions presented in this study are included in the article, further inquiries can be directed to the corresponding author.

## Ethics Statement

The animal study was reviewed and approved by Government of the state of Saxony-Anhalt according to the German guidelines for the care and use of animals in laboratory research (§8 TSchG; AZ: 42502-2-1585).

## Author Contributions

DK conducted DNA-methylation analysis and parts of the gene expression analysis and wrote significant parts of the manuscript including preparation of figures. CF conducted animal experiments including stress exposure, collected brain samples, and conducted parts of the gene expression analysis. NG was involved in the animal experiments, collected brain samples, and set up the DNA-methylation protocol. KB supervised the project, planned the experimental design, and wrote and edited significant parts of the manuscript and provided funding (DFG, BR 1692/12-1). JB supervised and planned the project and experimental design, was involved in the statistical analysis, wrote and edited large parts of the manuscript and provided funding (DFG, BO 1674/8-1). All authors contributed to the article and approved the submitted version.

## Conflict of Interest

The authors declare that the research was conducted in the absence of any commercial or financial relationships that could be construed as a potential conflict of interest.

## Publisher’s Note

All claims expressed in this article are solely those of the authors and do not necessarily represent those of their affiliated organizations, or those of the publisher, the editors and the reviewers. Any product that may be evaluated in this article, or claim that may be made by its manufacturer, is not guaranteed or endorsed by the publisher.

## Footnotes

1
www.psychologie.hhu.de/arbeitsgruppen/allgemeine-psychologie-und-arbeitspsychologie/gpower.html
